# The relationship between obsessive–compulsive symptoms and real-life functioning in schizophrenia: New insights from the multicenter study of the Italian Network for Research on Psychoses

**DOI:** 10.1192/j.eurpsy.2024.1747

**Published:** 2024-04-29

**Authors:** Matteo Tonna, Davide Fausto Borrelli, Eugenio Aguglia, Paola Bucci, Bernardo Carpiniello, Liliana Dell’Osso, Andrea Fagiolini, Paolo Meneguzzo, Palmiero Monteleone, Maurizio Pompili, Rita Roncone, Rodolfo Rossi, Patrizia Zeppegno, Carlo Marchesi, Mario Maj

**Affiliations:** 1 Department of Medicine and Surgery, Psychiatric Unit, University of Parma, Parma, Italy; 2 Department of Clinical and Molecular Biomedicine, Psychiatric Unit, University of Catania, Catania, Italy; 3 Department of Psychiatry, University of Campania Luigi Vanvitelli, Naples, Italy; 4 Section of Psychiatry, Department of Public Health, Clinical and Molecular Medicine, University of Cagliari, Cagliari, Italy; 5 Section of Psychiatry, Department of Clinical and Experimental Medicine, University of Pisa, Pisa, Italy; 6 Department of Molecular and Developmental Medicine, Division of Psychiatry, University of Siena, Siena, Italy; 7 Department of Neuroscience, Psychiatric Clinic, University of Padua, Padua, Italy; 8 Department of Medicine, Surgery and Dentistry “Scuola Medica Salernitana” Section of Neuroscience, University of Salerno, Salerno, Italy; 9 Department of Neurosciences, Mental Health and Sensory Organs, S. Andrea Hospital, University of Rome La Sapienza, Rome, Italy; 10 Unit of Psychiatry, Department of Life, Health and Environmental Sciences, University of L’Aquila, L’Aquila, Italy; 11 Section of Psychiatry, Department of Biotechnological and Applied Clinical Sciences, University of L’Aquila, L’Aquila, Italy; 12 Department of Translational Medicine, Psychiatric Unit, University of Eastern Piedmont, Novara, Italy

**Keywords:** disorganization, evolution, neurocognition, obsessive–compulsive disorder, ritual behavior, schizophrenia comorbidity, social cognition

## Abstract

**Background:**

Although obsessive–compulsive disorder (OCD) is highly prevalent in schizophrenia, its relationship with patients’ real-life functioning is still controversial.

**Methods:**

The present study aims at investigating the prevalence of OCD in a large cohort of non-preselected schizophrenia patients living in the community and verifying the relationship of OCD, as well as of other psychopathological symptoms, with real-life functioning along a continuum of OCD severity and after controlling for demographic variables.

**Results:**

A sample of 327 outpatients with schizophrenia was enrolled in the study and collapsed into three subgroups according to OCD severity (subclinical, mild–moderate, severe). A series of structural equation modeling (SEM) was performed to analyze in each subgroup the association of obsessive–compulsive symptoms with real-life functioning, assessed through the Specific Levels of Functioning Scale and the UCSD Performance-Based Skills Assessment. Moreover, latent profile analysis (LPA) was performed to infer latent subpopulations. In the subclinical OCD group, obsessive–compulsive symptoms (OCS) were not associated with functioning, whereas in the mild–moderate OCD group, they showed a positive relationship, particularly in the domains of work and everyday life skills. The paucity of patients with severe OCD did not allow performing SEM analysis in this group. Finally, LPA confirmed a subgroup with mild–moderate OCS and more preserved levels of functioning.

**Conclusions:**

These findings hint at a positive association between mild–moderate OCD and real-life functioning in individuals with schizophrenia and encourage a careful assessment of OCD in personalized programs to sustain daily life activities.

## Introduction

Schizophrenia is a heterogeneous neurodevelopmental disorder [1, 2], whose variety in clinical presentation lies in part on a complex interplay between different and quite distinct psychopathological dimensions [3]. Similarly, symptom dimensions seem to have a different impact on functional outcome, involving both illness and non-illness-related factors [4], through complex, probabilistic, nonlinear dynamics [5]. In order to advance knowledge on the relative role of the largest possible number of variables on real-life functioning in people with schizophrenia, the Italian Network for Research on Psychoses (NIRP) carried out a large multicenter study involving 921 community-dwelling, clinically stable patients with that diagnosis (Galderisi et al., 2020). In this regard, recent studies from the NIRP [4, 6] have confirmed that negative and disorganization dimensions are the major psychopathological determinants affecting real-life functioning and are the strongest predictors of poor functional outcome.

However, among psychopathological dimensions, obsessive–compulsive symptoms (OCS), which are reported in approximately 30% of patients with schizophrenia [7], have been found to exert an effect on functioning. In this regard, over the time, OCS have gone from playing an improving role to a worsening one. In fact, while OCS were initially viewed as a compensatory mechanism counteracting the deteriorating course of the illness [8–10], from the seminal work by Fenton and McGlashan (1986) [11], OCS started to be associated with a worse clinical outcome, greater disability, and poorer quality of life [12, 13].

Discrepancies among studies may have different methodological explanations:

a) The operational criteria used to disentangle true obsessions from “pseudo-obsessions” or delusional constructs. In fact, broader defined OCS might be less distinguishable from a wide range of basic psychotic phenomena, self-disorders (e.g., “hyperriflexivity”), stereotypies, or frank delusional beliefs (for a review, see Rasmussen and Parnas, 2022) [14].

b) The methodology used to approach OCS: that is, categorical versus dimensional model and the cut-offs of severity. Conflicting results might in fact reflect a differential effect of OCS depending on their severity. De Haan et al. (2013) [15] first hypothesized that subthreshold OCS exert an improving effect, whereas full-blown obsessive–compulsive disorder (OCD) a worsening one. In a similar vein, adopting a strict dimensional approach, a previous study [16] observed that the relationship between OCS and social functioning gradually shifted, along a severity continuum, from a positive association in patients with mild OCS to a negative one in those with more severe OCS. On the other hand, cluster analytic approaches (Lysaker et al., 2004; Swets et al., 2019) resulted in two groups with mild OCS severity, one with relatively good social functioning and one with relatively poor social functioning.

c) The inclusion of the effect of other clinical variables, such as disorganization and cognition, which can moderate the impact of OCS on functioning. For example, it has been found that OCS maintain an impact on functioning only in patients with mild disorganization symptoms, whereas their effect vanishes at higher severity of disorganization [17]. This complex interaction might also involve cognitive function. It has been suggested that OCS and disorganization symptoms offset each other in their effect on executive function in nonclinical population [18]. In clinical population, there is evidence for a differential effect of OCS (improving or worsening according to a severity gradient) on specific cognitive domains in both patients with schizophrenia [19, 20] and at-risk individuals [21]. Therefore, also for obsessive dimension, cognition might represent a crucial node, filtering the impact of OCS on functioning.

d) Finally, in previous studies, social functioning was evaluated as a unique construct, whereas real-life functioning is actually multifaceted, encompassing different domains (e.g., working skills, interpersonal relationship, community activities, and daily life abilities), which in turn are shaped by patients’ context-related variables and personal resources [6]. Therefore, the patterns of relationships among obsessive–compulsive and other symptom dimensions might vary according to the functional domain considered.

Considering all these methodological aspects, the present study was undertaken to: 1) assess the prevalence of OCS in a large cohort of non-preselected schizophrenia patients living in the community and 2) verify the pattern of associations between OCS; other symptoms (positive, negative, disorganization); cognitive dimensions (neuro and social cognition); and real-life functioning along a continuum of OCS severity and controlling for demographic variables (i.e., age, gender, years of illness, years of education).

This study, extending the findings of previous NIRP studies, adopted a dimensional approach to capture the heterogeneity in symptoms and real-life functioning expressed across individuals, as well as to grasp such non-prototypical (intermediate, mixed, or subthreshold) clinical configurations, hardly classified by categorical models [22, 23]. Moreover, we focused on specific functional domains (rather than assessing functioning globally) to test whether OCS had a differential relationship with the various areas of functioning.

Concerning OCS severity, a recent study [24] proposed a Y-BOCS total score of 13 as the threshold differentiating subclinical from clinical OCD, whose severity is mild to moderate for scores ranging from 14 to 29 and severe over 29. The authors proposed these cut-offs as benchmarks for OCD across lifespan, cultures, countries, and gender. Therefore, in the present study, these criteria were used to define OCD subgroups (subclinical, mild–moderate, severe).

## Methods*Study population*

The study was conducted in a large representative sample of outpatients with schizophrenia participating in the multicenter study of the Italian NIRP.

Inclusion criteria were: 1) a diagnosis of schizophrenia according to the Diagnostic and Statistical Manual of Mental Disorders - Fourth Edition (DSM-IV) and confirmed with the Structured Clinical Interview for DSM-IV – Patient version [25] and 2) an age between 18 and 66 years.

Exclusion criteria were: 1) a history of head trauma with loss of consciousness, 2) a history of moderate to severe intellectual disability or neurological diseases, 3) a history of alcohol and/or substance abuse in the last 6 months, and 4) treatment modifications and/or hospitalization due to symptom exacerbation in the last 3 months.

All patients signed a written informed consent to participate after receiving a comprehensive explanation of the study procedures and goals. Approval of the study protocol was obtained from the Local Ethics Committees of each participating center.

### Assessment

OCD severity was measured with the Yale-Brown Obsessive–Compulsive Scale (YBOCS) [26], a semi-structured interview that does not depend on specific types of symptoms (e.g., washing, checking), but on aspects of those symptoms as reported by the patient during the interview (e.g., duration, interference, degree of resistance). Recently, OCD severity cut-offs have been empirically defined as follows: 0–13 scores defined subclinical OCD, 14–21 scores mild OCD, 22–29 scores moderate OCD, and 30–40 scores severe OCD [24].

In the present study, we decided to follow a narrow definition of OCS, implying: 1) strictly egodystonic features (i.e., OCS with insight and not related to positive symptoms) [12]; 2) typical contents of OCD dimensions (e.g., contamination obsessions with washing and cleaning compulsions) [27]; and 3) typical motor pattern of OCD compulsions (i.e., rigid repetition of acts with redirection of attention on the performance itself) [28, 29].

The Positive and Negative Syndrome Scale (PANSS) [30] was used to rate symptom severity of positive and disorganization domains. We adopted the consensus five-factor solution proposed by Wallwork and colleagues (2012) [31], assessing positive symptoms using P1 (delusions), P3 (hallucinatory behavior), P5 (grandiosity), G9 (unusual thought content), and disorganization using P2 (conceptual disorganization), N5 (difficulty in abstract thinking), and G11 (poor attention).

Negative symptoms were assessed with the Brief Negative Symptom Scale (BNSS) [32], which includes 13 items, rated from 0 (normal) to 6 (most impaired), and five negative symptoms domains (anhedonia, asociality, avolition, blunted affect, and alogia).

Neurocognition was measured according to the six cognitive domains of the MATRICS Consensus Cognitive Battery (MCCB) [33, 34]: speed of processing, attention/vigilance, working memory, verbal learning, visual learning, reasoning, and problem solving.

Social cognition was measured using the Facial Emotion Identification Test (FEIT) [35], which examines emotion perception, and the Awareness of Social Inference Test (TASIT) [36], which is organized into three sections (Emotion Evaluation, Social Inference [Minimal] and Social Inference [Enriched]).

Functional capacity was assessed using the UCSD Performance-based Skills Assessment (UPSA) Brief [37], performance-based instrument that assesses “financial skills” and “communication skills,” while global functioning was evaluated using the Specific Levels of Functioning Scale (SLOF) [38]. Since our interest was to identify a model aimed at identifying the predictors of different aspects of functioning in schizophrenia, we did not used the overall composite score but only the three SLOF-domains (working abilities, interpersonal relationships, and everyday life skills), that are the most informative for patients with schizophrenia.

All scales exhibited adequate to good internal consistency: YBOCS (α = 0.96), PANSS Positive symptoms (α = 0.78), PANSS Disorganization (α = 0.63), BNSS (α = 0.96), UPSA (α = 0.88), SLOF working abilities (α = 0.92), SLOF interpersonal relationship (α = 0.89), and SLOF everyday life skills (α = 0.94).

### Statistical analyses

#### Software

All statistical analyses were performed using R for statistical computing (version 3.6.1, open source, available at https://www.r-project.org/).

#### Clinical features

First, descriptive statistics were used to examine the sociodemographic and clinical characteristics of the whole sample, including the frequency of OCD.

#### OCD groups

According to the recent cut-offs of OCD severity proposed by Cervin and colleagues [24], and in line with our previous finding of a shift in OCS-functioning relationship from direct to inverse at a YBOCS value of 13 [16], we collapsed the study sample into three subgroups along a gradient of OCD severity (subclinical, mild–moderate, and severe OCD groups). However, as subclinical group also included individuals with no-OCS, we verified whether levels of functioning domains differentiated individuals with minimal OCS (YBOCS = 1–13) from those with no-OCS.

We chose to merge mild and moderate OCS into a unique subgroup in order to create subgroups with adequate sample size to perform statistical analyses.

#### Structural equation modeling

To make inferences about the relationship between symptom dimensions and global functioning, we fitted regression models within a structural equation modeling (SEM) framework, using the R library *lavaan.* The SEM tests the relationship between variables by means of simultaneous confirmatory factor analysis and regression analysis. Such an approach permits the verification of the appropriateness of predicted relationships or models, which allows complex relationships between variables [39, 40]. We used the *lavaan* “orthogonal” function to set to zero all covariances among latent variables. Moreover, we used the *lavaan* “std.lv” function to determine the metric of each latent variable by fixing their (residual) variances to 1.0.

We, therefore, assumed a model using the various dimensions of functioning (i.e., UPSA, SLOF interpersonal relationships, SLOF everyday life skills, and SLOF work skills) as dependent variables. To do this, we assumed the organization of the independent manifest variables according to latent factors (i.e., positive symptoms, negative symptoms, disorganization, social cognition, neurocognitive functioning, and demographic features). This approach is preferred since manifest variables might be imperfect measurements of a single underlying concept [41]. In this direction, positive symptoms were defined as a latent construct based on the consensus factor solution proposed by Wallwork et al. 2012 [31], including P1(delusions), P3 (hallucinatory behavior), P5 (grandiosity), and G9 (unusual thought content), while the latent construct of disorganization was defined using three items of the PANSS scale: P2 (conceptual disorganization), N5 (difficulty in abstract thinking), and G11 (poor attention). Negative symptoms latent factor was determined by using the five BNSS domains: anhedonia, asociality, avolition, blunted affect, and alogia. Social cognition was defined as a latent construct based on the three TASIT and FEIT scales. Finally, to define the neurocognitive latent construct, we used the six cognitive domains of the MCCB. Since Y-BOCS items are indicators of OCS severity and not of symptoms heterogeneity, we have retained the overall Yale-Brown score as a manifest variable. Finally, we also considered as manifest variables different demographic features (i.e., age, years of illness, years of education, and gender). Thus, we accounted for covariance among symptom dimensions, resulting in unique associations between each psychopathological variable and the dependent variables.

Finally, to address the role of OCS on functioning, the SEM was performed in subclinical, mild–moderate and severe OCD groups. However, as subclinical group also included individuals with no-OCS, we also performed an SEM analysis in individuals with minimal OCS (YBOCS = 1–13).

The goodness-of-fit indices are a critical question in almost every application of SEM and should be considered before interpreting the results [42]. As recommended, several fit indices were examined to evaluate model fit: confirmatory fit index (CFI; adequate fit indicated by >0.90), standardized root mean square residual (SRMR, adequate fit indicated by <0.8), and root mean square error of approximation (RMSEA). Recommendations for RMSEA cut-off points have undergone a number of changes in the last decades (for a summary, see Hooper et al. 2008) [43]. However, a cut-off value close to. 06 [44] or a stringent upper limit of 0.07 [45] seems to be the general consensus in this area [43].

#### Latent profile analysis

Finally, we performed a latent profile analysis (LPA) to better infer latent subpopulations. In fact, LPA allows to type participants with varying degrees of probability into subgroups with different attributes [46]. Differently from latent class analysis, LPA can identify latent profiles based on responses to several continuous (and not categorical) indicators. We used the *mclust* R package, which is based on parameterized finite Gaussian mixture models. To avoid a large number of indicators, in contrast to SEM, we used the sums of the psychopathological variables as well as neurocognitive and social cognition variables. Thus, each participant was assigned a probability for each of the estimated subpopulation, based on their pattern of scores on the indicators considered. We run consecutive models with increasing numbers of classes (G = 1 to G = 9). For each model, we let the package fit four model variants (“EEI,” “EEE,” “VVI,” and “VVV”) and select the best-fitting one. We followed the suggestions by Spurk and colleagues [46] for a comprehensive representation of the results.

## Results

### Sample characteristics

The study sample comprised 327 patients with a mean age of 45.8 ± 10.4 years. They were predominantly males (*n* = 231, 70.6%) and with an average years of education of 11.7 ± 3.4 years.

Their mean age at onset of schizophrenia was 23.5 ± 6.9 years with a mean illness duration of 22.3 ± 10.4 years.

### OCD groups

The average of Y-BOCS scores in the whole sample were 5.7 ± 8.1 for the total score, 3.1 ± 4.4 for the obsessive score and 2.6 ± 4.1 for the compulsive score. Subclinical OCD group comprised 231 patients (70.6%), whereas mild–moderate OCD group included 87 subjects (26.6%) (mild OCD, *n* = 63; moderate OCD, *n* = 24), and severe OCD group 9 (2.7%). Sociodemographic, psychopathological, and clinical variables of these groups are reported in Table 1. Data on the group with severe OCD were not reported given the paucity of the sample. In the study sample, 138 patients (42.2%) reported no OCS (YBOCS = 0), in line with the non-preselected characteristic of the sample. These patients were included in the subclinical OCD group in order not to dichotomize the sample, according to the strict dimensional model by Cervin and colleagues [24]. It should be noted, however, that in the subclinical group, the levels of functioning were not influenced by individuals with no-OCS, since individuals with no-OCS and with minimal OCS (YBOCS score 1–13) showed similar levels of functioning: SLOF-w: no-OCS = 20.2 ± 16.17, OCS 1–13 = 19.9 ± 6.2, (t = 0.38, *p* = .70); SLOF-e: no-OCS = 45.5 ± 9.31, OCS 1–13 = 45.0 ± 9.1 (t = 0.37, *p* = .71); SLOF-: no-OCS = 23.0 ± 5.94, OCS 1–13 = 22.5 ± 5.84 (t = 1.03, *p* = .14); and UPSA: no-OCS = 14.6 ± 4.9, OCS 1–13 = 14.0 ± 4.81 (t = 0.91, *p* = .36).

**Table 1.** Sociodemographic and clinical features in the total sample and in the subclinical and mild–moderate OCD subgroups

	Subclinical OCD group(*n* = 231)	Mild–moderate OCD group(*n* = 87)	Total sample(*n* = 327)	Comparisonbetween groups	
	28.5	33	29.35	χ^2^ = 0.49	*p* = 0.48
Females (%)	Mean (SD)	Mean (SD)	Mean (SD)	t	*p*
Age	46.57 (±10.52)	43.66 (±9.96)	45.86 (±10.41)	2.28	0.02*
Age of onset	23.56 (±7.07)	23.06 (±6.47)	23.51(±6.99)	0.59	0.55
Years of education	11.38 (±3.49)	12.91 (±3.42)	11.78 (±3.49)	−3.52	0.001**
Years of illness	23.01 (±10.81)	20.60 (±9.41)	22.35 (±10.45)	1.95	0.05*
PANSS P1 (delusions)	2.33 (±1.58)	2.4 (±1.51)	2.38 (±1.59)	−0.33	0.73
PANSS P2 (conceptual disorganization)	2.53 (±1.54)	2.25 (±1.36)	2.46 (±1.50)	1.57	0.12
PANSS P3 (hallucinatory behavior)	1.82 (±1.32)	1.87 (±1.37)	1.84 (±1.33)	−0.27	0.78
PANSS P5 (grandiosity)	1.56 (±1.12)	1.40 (±0.89)	1.53 (±1.08)	1.32	0.18
PANSS N5 (difficulty in abstract thinking)	3.25 (±1.66)	3.03 (±1.61)	3.20 (±1.66)	1.05	0.29
PANSS G9 (unusual thought content)	2.37 (±1.51)	2.62 (±1.53)	2.46 (±1.55)	−1.29	0.19
PANSS G11 (poor attention)	2.40 (±1.39)	2.27 (±1.25)	2.36 (±1.36)	0.77	0.43
BNSS anhedonia	7.45 (± 4.65)	7.12 (± 4.09)	7.38 (±4.49)	0.61	0.54
BNSS asociality	5.52 (±3.19)	5.28 (±2.96)	5.51 (±3.14)	0.63	0.52
BNSS avolition (apathy)	5.12 (±3.01)	4.86 (±2.93)	5.06 (±3.01)	0.71	0.47
BNSS distress	1.95 (±1.60)	2.01 (±1.54)	1.99 (±1.60)	−0.27	0.78
BNSS blunted affect	7.84 (±4.69)	7.0 (±4.61)	7.66 (±4.72)	1.44	0.15
BNSS alogia	4.73 (±3.56)	3.50 (±3.28)	4.41 (±3.55)	2.9	0.004**
MCCB speed of processing	32.30 (±12.88)	35.88 (±11.06)	33.68 (±11.52)	−2.45	0.01*
MCCB attention/vigilance	35.54 (±15.41)	35.96 (±13.45)	36.44 (±12.73)	−0.24	0.81
MCCB working memory	2.33 (±1.58)	2.40 (±1.51)	35.46 (±11.74)	−1.22	0.22
MCCB verbal learning	37.31 (±11.39)	38.01 (±12.56)	36.86 (±11.41)	−0.45	0.65
MCCB visual learning	30.97 (±14.64)	33.50 (±10.94)	33.45 (±12.79)	−1.66	0.09
MCCB reasoning and problem solving	38.37 (±10.31)	40.08 (±9.29)	39.56 (±9.78)	−1.41	0.15
TASIT Sect. 1 (correct items)	19.45 (±5.91)	22.37 (±4.57)	21.30 (±4.72)	−4.66	0.001**
TASIT Sect. 2 (correct items)	39.38 (±12.39)	43.73 (±12.35)	39.31 (±11.21)	−2.79	0.005**
TASIT Sect. 3 (correct items)	41.32 (±11.82)	41.37 (±10.59)	38.75 (±10.88)	−0.04	0.96
FEIT (correct responses)	38.45 (±9.83)	38.60 (±9.15)	37.96 (±8.69)	−0.13	0.89
UPSA	14.39 (±5.09)	15.71 (±4.43)	14.44 (±4.93)	−2.26	0.02*
SLOF activities	45.35 (±9.81)	47.12 (±7.48)	45.77 (±9.32)	−1.71	0.08
SLOF work	20.10 (±6.14)	19.59 (±6.3)	19.92 (±6.17)	0.64	0.52
SLOF interpersonal relationships	22.46 (±5.84)	22.32 (±6.22)	22.39 (±5.92)	0.18	0.85

Abbreviations: BNSS, Brief Negative Symptom Scale; FEIT, Facial Emotion Identification Test; MCCB, Matrics Consensus Cognitive Battery; PANSS, Positive and Negative Syndrome Scale; SLOF, Specific Levels of Functioning; TASIT, The Awareness of Social Inference Test; UPSA, Performance-Based Skills Assessment Brief; Y-BOCS, Yale-Brown Obsessive–Compulsive Scale.

*Note*: Total sample comprised also nine patients with severe OCD. The significant differences are indicated with stars.

* *p* < .05;

** *p* < .01.

### Antipsychotic treatment

Almost all subjects were on antipsychotic treatment (99.4%; 24.8% on first-generation antipsychotics; 72.8% on second-generation antipsychotics, while for 2.4% no information was available). Eighty-one patients (24.8%) were treated with clozapine. The mean daily clozapine dose was 283.4 ± 163.1 mg. To control for the potential effect of clozapine on the OCS severity (for a summary, see Schirmbeck and Zink 2012) [47], we first performed a nonparametric correlation in this subsample which showed no relationship between clozapine dose and OC symptoms severity (r = .114, *p* = .384). We then performed a *t* test in which no significant differences were found between the patients in treatment with clozapine and the others in terms of OCS severity (t = .626, *p* = .532).

Polypharmacy was reported by 23.2% of patients (3.1% of patients were treated with a combination of three different antipsychotics; none of those were in treatment with clozapine). At least one relapse was reported in 39.1% of the participants during the last year; among them, the median number of relapses was 2 and the median number of hospitalizations was 1.

### Relationship between OCD and functioning

SEM was performed in the subclinical and mild–moderate OCD groups.

**Figure 1.**
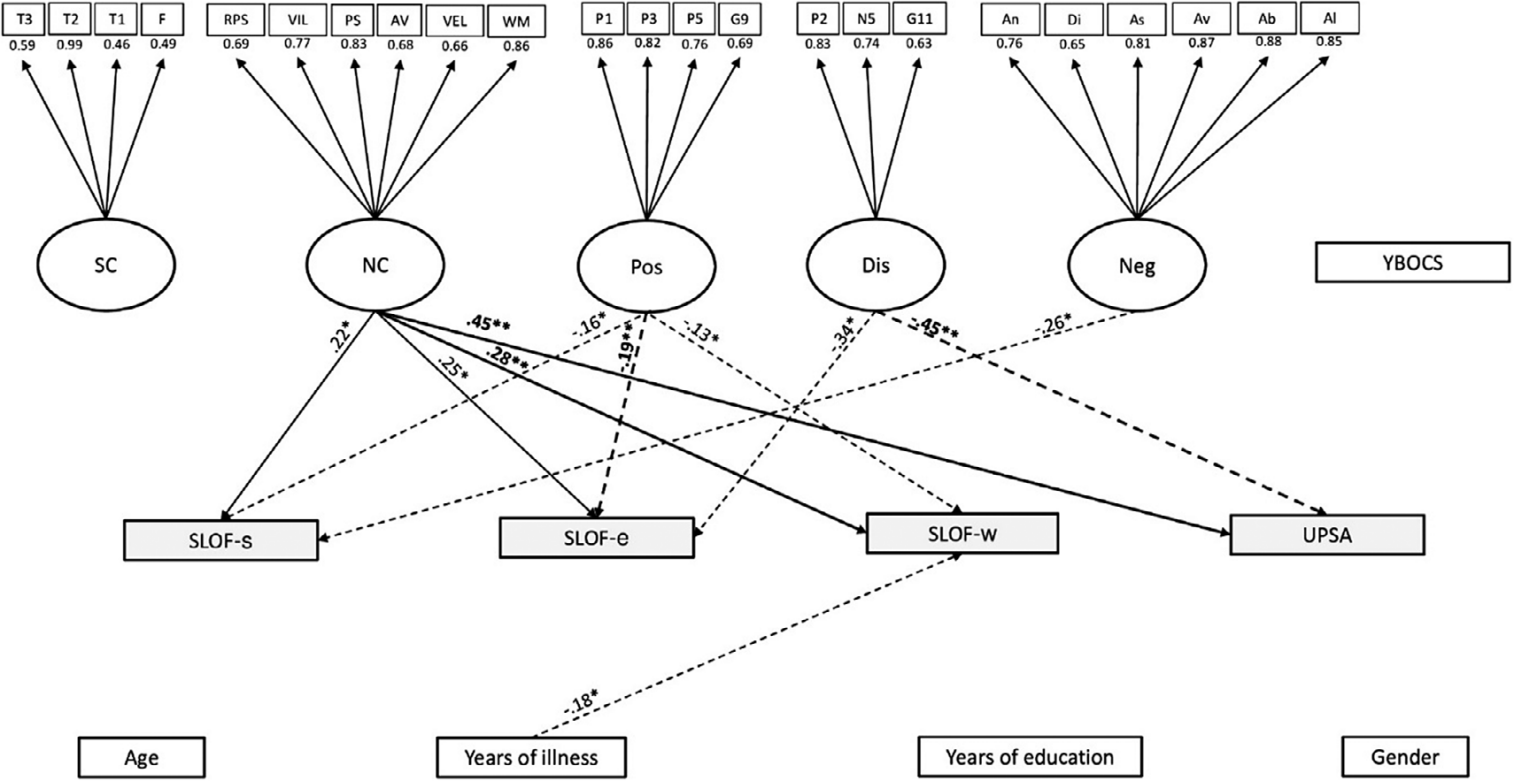
Structural equation modeling (SEM) in the subclinical OCD subgroup. The rectangles represent observed variables. The squares represent indicators for the latent variables (circles). The arrows represent the paths. T3 = Awareness of Social Inference Test 3, T2 = Awareness of Social Inference Test 2, T1 = t Awareness of Social Inference Test 1, F=Facial Emotion Identification Test, =RPS = reasoning and problem solving, VIL = visual learning, PS = speed of processing, AV = attention/vigilance, VEL = verbal learning, WM = working memory, P1 = delusions, P3 = hallucinatory behavior, P5 = grandiosity, G9 = unusual thought content, P2 = conceptual disorganization, N5 = difficulty in abstract thinking, G11 = poor attention, An = anhedonia, Di = distress, As = asociality, Av = avolition, Ab = blunted affect, Al = alogia, Age = age, YI = years of illness, SC = social cognition, NC = neurocognition, Pos = positive symptoms, Dis = disorganization, Neg = negative symptoms, Demo = demographic features, YBOCS=Yale-Brown assessment scale, W = work skills (SLOF-w), S = interpersonal relationships (SLOF-s), E = everyday skills (SLOF-e), UPSA = Performance-based Skills Assessment Brief. The associations between dependent and independent variables are expressed through the standardized estimates, based on variances of both observed and latent variables. The significant associations are indicated with stars (*p < .05; **p < .01). Negative effects are indicated with dashed arrows, positive effects with continuous arrows.

The model/data fit was adequate in the subclinical OCD group (*n* = 231; CFI = 0.9, SRMR = 0.1, RMSEA = 0.06). Work skills (SLOF-w) were predicted positively by neurocognition (*p* = .003) and negatively by positive symptoms (*p* = .03) and years of illness (*p* = .02). Everyday life skills (SLOF-e) were predicted positively by neurocognition (*p* = .012) and negatively by positive symptoms (*p* = .002) and disorganization (*p* = .01). Interpersonal functioning (SLOF-s) was positively predicted by neurocognition (*p* = .018) and negatively predicted by positive and negative symptoms (respectively, *p* = .013 and *p* = .016). Functional capacity (UPSA) was positively predicted by neurocognition (*p* = .001) and negatively by disorganization (*p* = .001). The model/data is reported in Figure 1.

As subclinical group also included individuals with no-OCS and with minimal OCS (YBOCS = 1–13), we also performed an SEM analysis only in patients with minimal OCS, which failed to find a relationship between OCS severity and different domains of functioning: YBOCS scores on SLOF-W *p* = .50; SLOF-E *p* = .88; SLOF-S *p* = .51; and UPSA *p* = .42.

**Figure 2.**
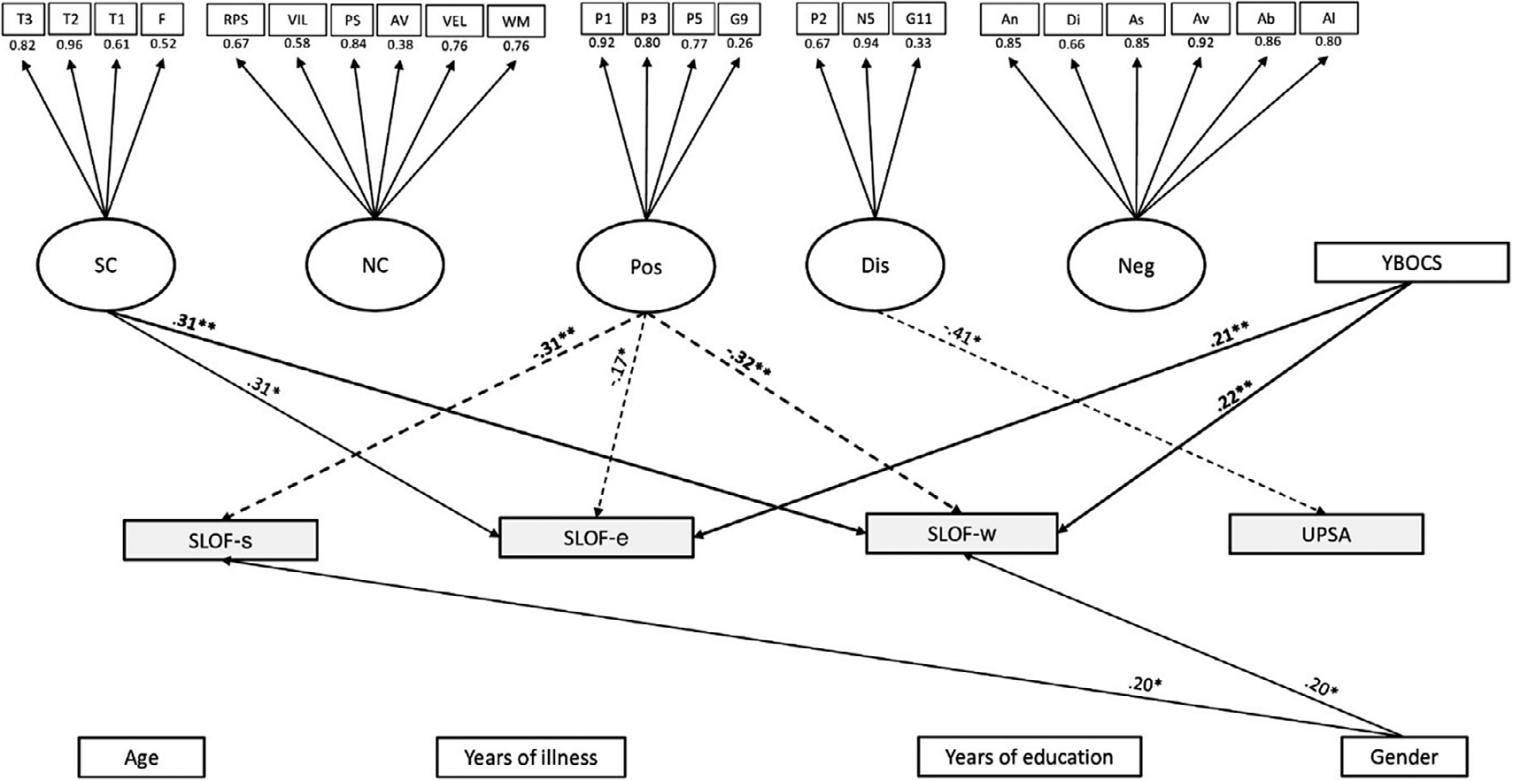
Structural equation modeling (SEM) in the mild–moderate OCD subgroup. The rectangles represent observed variables. The squares represent indicators for the latent variables (circles). The arrows represent the paths. T3 = Awareness of Social Inference Test 3, T2 = Awareness of Social Inference Test 2, T1 = t Awareness of Social Inference Test 1, F=Facial Emotion Identification Test, RPS = reasoning and problem solving, VIL = visual learning, PS = speed of processing, AV = attention/vigilance, VEL = verbal learning, WM = working memory, P1 = delusions, P3 = hallucinatory behavior, P5 = grandiosity, G9 = unusual thought content, P2 = conceptual disorganization, N5 = difficulty in abstract thinking, G11 = poor attention, An = anhedonia, Di = distress, As = asociality, Av = avolition, Ab = blunted affect, Al = alogia, Age = age, YI = years of illness, SC = social cognition, NC = neurocognition, Pos = positive symptoms, Dis = disorganization, Neg = negative symptoms, Demo = demographic features, YBOCS=Yale-Brown assessment scale, W = work skills (SLOF-w), S = interpersonal relationships (SLOF-s), E = everyday skills (SLOF-e), UPSA = Performance-based Skills Assessment Brief. The associations between dependent and independent variables are expressed through the standardized estimates, based on variances of both observed and latent variables. The significant associations are indicated with stars (*p < .05; **p < .01). Negative effects are indicated with dashed arrows, positive effects with continuous arrows.

The model/data fit was adequate in the mild–moderate OCD group (*n* = 87; CFI = 0.9, SRMR = 0.08, RMSEA = 0.07). Work skills (SLOF-w) were predicted positively by OCD severity (*p* = .004) and social-cognition (*p* = .006), and negatively by positive symptoms (*p* = .001). Everyday life skills (SLOF-e) were predicted positively by OCD severity (*p* = .009), social-cognition (*p* = .01), male gender (*p* = .034), while negatively predicted by positive symptoms (*p* = .027). Interpersonal functioning (SLOF-s) was positively predicted by male gender (*p* = .034) and negatively by positive symptoms (*p* = .002). Functional capacity (UPSA) was negatively predicted by disorganization (*p* = .001). The model/data is reported in Figure 2.

In severe OCD group, we did not conduct SEM analysis due to the paucity of patients. However, in this group, we performed a linear regression (univariate) analysis that showed a negative relationship between Y-BOCS total score and SLOF-e (β = −9.33, *p* = 0.007), SLOF-w (β = −1.66, *p* = 0.45), and SLOF-s (β = −1.91, *p* = 0.35).

### Latent profile analysis

**Figure 3.**
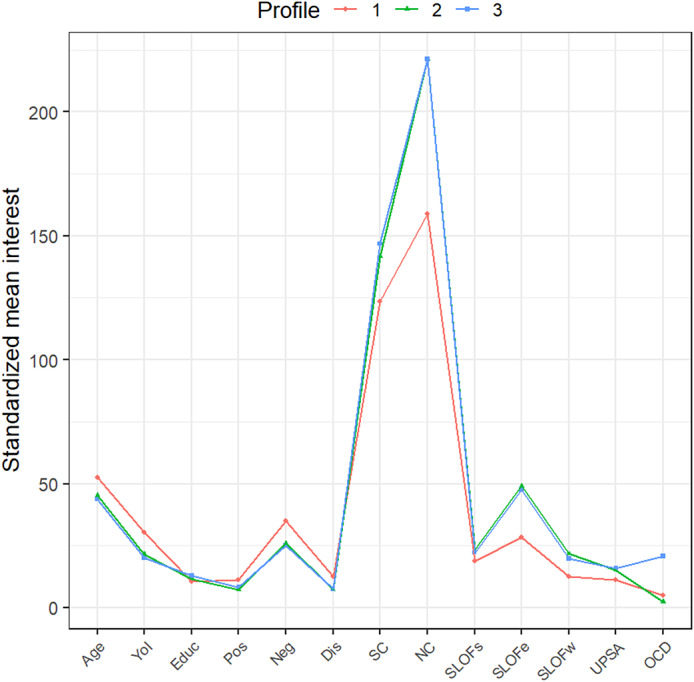
Latent profiles plot of the estimated means with point sizes proportional to the estimated mixing probabilities. YoI = Years of illness; Educ = Years of education; Pos = positive symptoms assessed by Positive and Negative Syndrome Scale; Neg = negative symptoms assessed by Brief Negative Symptom Scale; Dis = disorganization symptoms assessed by Positive and Negative Syndrome Scale; SC = social-cognition, expressed by the sum of Awareness of Social Inference Test domains and Facial Emotion Identification Test; NC = neurocognition expressed by the sum of MATRICS Consensus Cognitive Battery domains; SLOFs = interpersonal relationships assessed by Specific Levels of Functioning Scale; SLOFe = everyday skills assessed by Specific Levels of Functioning Scale; SLOFw = work skills assessed by Specific Levels of Functioning Scale; UPSA = functional capacity assessed by Performance-based Skills Assessment Brief; OCD = OCD symptoms severity assessed by Yale Brown Obsessive–Compulsive Scale.

We found that the best model was a three-class model with an EEE configuration (i.e., in which indicator variables are set to have zero covariances within and across classes) and a BIC of −29436.04. The three extracted profiles are represented in a latent profiles plot of the estimated means with point sizes proportional to the estimated mixing probabilities (Figure 3).

Individuals in the first extracted profile (Profile 1, *n* = 43, 13.15% of our sample) were older (52.42) with a higher duration of illness (30.48) and lower years of education (10.39) than other profiles. As to symptom severity, they had higher scores in positive symptoms (11.30), negative symptoms (35.09), and disorganization (12.49) than other profiles. Moreover, they showed lower levels in the four functioning indicators (SLOF-w = 12.57, SLOF-e = 28.23, SLOF-s = 18.66, UPSA = 11.22), in social cognition (123.46), and neurocognition (158.78) measures than the other profiles. In this group, the average of OCD severity was 4.96.

The second extracted profile included more than half of the participants (Profile 2, *n* = 192, 58.71% of our sample). Individuals in this profile were characterized by younger age (45.38), lower years of illness (21.48), and higher levels in years of education (11.57) than Profile 1. They had lower scores in positive symptoms (7.35), negative symptoms (25.94), and disorganization (7.24) than profile 1. Moreover, Profile 2 had higher levels in the four functioning indicators (SLOF-w = 21.74, SLOF-e = 49.07, SLOF-s = 23.27, UPSA = 15.10), in social cognition (141.82), and in neurocognition (221.17) measures than Profile 1. In this group, the average of OCD severity was 2.39.

The third profile (Profile 3, *n* = 92, 28.14% of our sample) was characterized by younger age (43.72), lower years of illness (20.24), and higher levels in years of education than Profiles 1 and 2. Moreover, individuals in Profile 3 had lower scores in positive symptoms (8.33) and disorganization (7.47) than Profile 1, and lower scores in negative symptoms (25.18) than Profiles 1 and 2. Regarding functioning indicators, the third Profile showed higher scores in SLOF outcomes (SLOF-w = 19.71, SLOF-e = 47.45, SLOF-s = 22.26) than Profile 1 and comparable than Profile 2. Moreover, Profile 3 had higher score in functional capacity (UPSA = 15.91), as well as higher scores in social cognition (146.8) and neurocognition (221.29) than the other profiles. In this group, the average of OCD severity was 20.78.

## Discussion

The present study was aimed to assess the prevalence of OCD in a large sample of individuals with schizophrenia living in a community and to verify the patterns of associations between OCS, main symptom, cognitive dimensions, and real-life functioning, according to the OCS severity gradient and controlling for demographic variables. Several interesting results were found in this study.

First, we confirmed the high prevalence of OCS in schizophrenia individuals: 96 out of 327 patients (28.4%) had clinically significant OCD, according to recently proposed cut-offs [24]; among them, 87 patients (26.6%) had mild–moderate OCD, whereas 9 patients (2.7%) presented severe OCD. These results are in line with the existing literature: approximately 30% of schizophrenia individuals show clinically significant OCS according to the meta-analysis by Swets and colleagues [7]. Remarkably, the occurrence of OCS in our sample was not related to clozapine treatment; therefore, a significant “pro-obsessive” effect of clozapine [47] on OCS prevalence rate may be ruled out in this study. The raw data in itself confirm that OCS represent a non-negligible dimension in schizophrenia and raises important questions about their clinical implications, in particular their relationship with social functioning.

Second, in patients with subclinical OCD, we failed to find a relationship between OCS and global functioning. We can rule out that this result might be due to individuals with no-OCS (which were included in this group) since we failed to find significant differences in functioning between individuals without OCS and with minimal OCS. Furthermore, in patients with minimal OCS, OCS severity was not related to functioning.

On the contrary, both neurocognition and symptom dimensions (i.e., positive, negative and disorganization) were differently associated with the main functional domains. Specifically, neurocognition predicted work skills (SLOF-w), everyday life skills (SLOF-e), interpersonal relationships (SLOF-s), and functional capacity (UPSA). The role played by neurocognition was in concert with that of psychopathological dimensions, which were differentially related to single functional subdomains with a prominent role of disorganization on functional capacity. These results confirm previous findings from NIRP studies, namely the crucial role of neurocognition associated with symptom dimensions, in particular disorganization [4, 6, 48], on patients’ real-life functioning. Although discrete dimensions, both disorganization and neurocognition share a partly overlapping pathophysiology, lying on an impaired integration of contextual information [49]. This would support the classical view of a fundamental structural disaggregation/dissociation (*Spaltung*) [50] or discordance [51] in the formal modes of consciousness as the core feature of schizophrenia [52].

Third, in the subgroup of patients with mild–moderate OCD, the pattern of associations among psychopathological variables, cognition, and main functioning domains substantially changed, with a prominent and positive role of OCS on functioning in concert with social cognition. In fact, OCS severity positively predicted both work (SLOF-w) and everyday life skills (SLOF-e). That is, the more symptomatic the patients were in their OCS, the more preserved real-life functioning was in important areas such as vocational performances (e.g., employable skills, level of supervision required to complete tasks, ability to stay on task, punctuality) and everyday activities (e.g., household activities, handling of personal finances and use of the telephone or public transportation). On the contrary, no associations were found between OCS and both interpersonal functioning (SLOF-s) and functional capacity (UPSA). We speculate that the repetitious and ritualized behavioral patterns induced by OCS (at a mild–moderate level) may confer order and stability over specific functional domains, thus reducing the functional impairment associated with schizophrenia [53]. In particular, vocational and daily life activities might be more sensitive to the OCS “ordering” effect, since they are mainly shaped by habitual/routinized behaviors, of which OCS would represent the psychopathological counterpart [54, 55]. Interpersonal functioning (e.g., initiating, accepting and maintaining social contacts) and functional capacity (i.e., the ability to perform tasks relevant to everyday life in a structured environment) would escape such an effect since, we suggest, they are inherently linked to the core of schizophrenia psychopathology.

Instead, the major role of social cognition over neurocognition in this subgroup of patients was unexpected. Overall, the mild–moderate OCD group showed higher levels of social cognition (as well as of speed of processing) than the subclinical OCD group. This may be partly because individuals in the mild–moderate OCD group were also younger, with higher years of education and lower duration of illness than subjects in the subclinical OCD group, and thus more likely to have preserved abilities in the social cognition domain. It should be noted, however, that the two groups did not differ in the levels of the main functioning domains. Therefore, sociodemographic differences may explain the cognitive profile of these patients more than their functional levels and the association patterns between the latter and OCS.

These findings were confirmed after performing an LPA in the whole sample. In fact, the third profile extracted (Profile 3), which was characterized by a mild–moderate OCD severity (average YBOCS score = 20.78), showed higher levels in functioning domains than Profile 1 and comparable levels than Profile 2, both of which had subclinical OCD severity (average YBOCS scores of 4.96 and 2.39, respectively).

Altogether, the findings of the present study would confirm the hypothesis of a positive relationship between mild OCS and specific functional domains [16, 17]. In previous studies, however, such a positive association with functioning was present in subclinical OCD, while in the present study, it appears in mild–moderate OCD. The discrepancy may be due to the different study samples: a small preselected sample of schizophrenia patients with OCS was evaluated in the previous studies, whereas a large cohort of non-preselected community-living individuals with schizophrenia was evaluated in the present one.

The positive association between mild OCS and functioning was questioned by Swets and colleagues [56], which failed to find evidence for a better prognosis in schizophrenia patients with mild OCS over 3 years. However, it should be noted that in this study, the follow-up investigation focused exclusively on the stability of negative symptoms, with the finding that only in the group of patients with mild OCS and poorer functioning, negative symptom severity remained higher over time. The poor prognosis in these patients may be explained by the fact they actually had higher primary, enduring negative symptoms (termed “deficit symptoms”), which in fact are stable over time and represent the major determinants of poorer functioning [57–59]. Moreover, in this study, disorganization symptoms were not investigated, thus not taking into account the moderation role of this dimension in the relationship between OCS and functioning, as previously found [17]. Finally, it should be noted that in previous studies, social functioning was assessed as a unique construct, whereas in the present study a multifaceted real-life functioning was considered.

From an evolutionary perspective, the positive association between mild/moderate OCS and functioning might be linked with the adaptive significance of ritual behavior throughout evolution, namely that of coping with unpredictability conditions [60] or “high-entropy” states [61]. It should be noted that OCD and ritual behavior share homologous features in terms of face validity (i.e., same formal structure) [62], construct validity (i.e., same neurobiological underpinnings, lying in the basal ganglia structures) [55], and predictive validity (as shown by robust animal models of OCD) [63]. In schizophrenia patients, the same “homeostatic” mechanism would be at work, with a superimposed “ordering” obsessive–compulsive structure over high-entropy/ unpredictability states due to the disorganizing process of psychosis [28, 29]. Therefore, the frequent occurrence of mild–moderate OCS in schizophrenia, probably underpinning a fronto-striatal dysconnectivity [64], ultimately results in more stable syndromic configurations, which allow the patient to preserve specific functional domains in the real life [65].

Fourth, the paucity of patients with severe OCD (*n* = 9; 2.7%) did not allow to perform SEM analysis in this group. Nevertheless, in these few patients, OCD severity showed a negative relationship with functioning in daily life activities. This finding, though preliminary, would, however, confirm the inverse association between severe OCS on functioning in individuals with schizophrenia found in previous studies [16]. Overall, the relationship between OCS and functioning seems to change dynamically along a severity gradient: from no association at all for subclinical OCD, to a positive association for mild–moderate OCD, up to a negative one for severe OCD.

To the best of our knowledge, this is the first study to evaluate the differential relationship of narrowly defined OCD, along a severity continuum, in a large cohort of non-preselected schizophrenia patients living in the community.

The present results should be viewed with the caveat of the following limitations. First, the cross-sectional design of the study cannot rule out the possibility that the relationship between OCD and other psychopathological variables may change over time or have a phase-dependent effect. Therefore, longitudinal studies are needed to confirm our results. Second, the patients enrolled in the study were outpatients with stable symptoms, thus not representative of patients in acute phases or in other clinical settings. Third, the limited number of patients with severe OCS did not allow investigating the relationship between OCS and functioning in this subgroup of patients. In a similar vein, the limited number of patients with mild/moderate OCS led to a reduction in model fit indices in this subgroup. Fourth, the current study did not examine the influence of past or current psychotherapeutic, medical, or psychosocial interventions; nor was the effect of polypharmacy controlled. Finally, the possible beneficial effects of serotoninergic drugs on OCS severity in the study sample were not taken into account.

Despite these limitations, the study also has important strengths. First, the large sample size. Second, the naturalistic design without selection bias related to randomized controlled designs and the statistical analysis. Third, the use of state-of-the-art instruments to assess real-world functioning, psychopathological variables, neurocognition, and social cognition.

## Conclusions

The results of the present study hint at a positive association between mild–moderate OCD and levels of work and daily life activities in schizophrenia. Since OCD may occur long before the clinical onset [65–67], future research should be addressed to investigate their effect in shaping the course and functioning in prepsychotic phases. Moreover, the present study encourages a careful assessment of OCS in individuals with schizophrenia in the search for targeted therapeutic and rehabilitation interventions to improve real-life functioning.
